# Analysis of Epigenetic Changes in Vitamin D Pathway Genes in Rheumatoid Arthritis Patients

**DOI:** 10.15388/Amed.2021.29.1.7

**Published:** 2022-07-26

**Authors:** Eglė Puncevičienė, Justina Gaiževska, Rasa Sabaliauskaitė, Kristina Šnipaitienė, Lina Vencevičienė, Dalius Vitkus, Sonata Jarmalaitė, Irena Butrimienė

**Affiliations:** Clinic of Rheumatology, Orthopaedics Traumatology and Reconstructive Surgery, Institute of Clinical Medicine of the Faculty of Medicine, Vilnius University, Vilnius, Lithuania;; State Research Institute Centre for Innovative Medicine, Vilnius, Lithuania;; Center of Rheumatology, Vilnius University Hospital Santaros klinikos, Vilnius, Lithuania; Institute of Biosciences, Life Sciences Center, Vilnius University, Vilnius, Lithuania;; National Cancer Institute, Vilnius, Lithuania; National Cancer Institute, Vilnius, Lithuania; Institute of Biosciences, Life Sciences Center, Vilnius University, Vilnius, Lithuania;; National Cancer Institute, Vilnius, Lithuania; Clinic of Internal Medicine, Family Medicine and Oncology, Faculty of Medicine, Vilnius University, Vilnius, Lithuania;; Center of Family Medicine, Vilnius University Hospital Santaros Klinikos, Vilnius, Lithuania; Institute of Biomedical Sciences of the Faculty of Medicine, Vilnius University, Vilnius, Lithuania;; Center of Laboratory Medicine, Vilnius University Hospital Santaros klinikos, Vilnius, Lithuania; Institute of Biosciences, Life Sciences Center, Vilnius University, Vilnius, Lithuania;; National Cancer Institute, Vilnius, Lithuania; Clinic of Rheumatology, Orthopaedics Traumatology and Reconstructive Surgery, Institute of Clinical Medicine of the Faculty of Medicine, Vilnius University, Vilnius, Lithuania;; State Research Institute Centre for Innovative Medicine, Vilnius, Lithuania;; Center of Rheumatology, Vilnius University Hospital Santaros klinikos, Vilnius, Lithuania

**Keywords:** DNA methylation, Rheumatoid arthritis, Vitamin D

## Abstract

**Background:**

Rheumatoid arthritis (RA) is an autoimmune inflammatory disease with complex etiopathogenesis launched by multiple risk factors, including epigenetic alterations. RA is possibly linked to vitamin D that is epigenetically active and may alter DNA methylation of certain genes. Therefore, the study aimed to evaluate the relationship between DNA methylation status of vitamin D signaling pathway genes (*VDR, CYP24A1, CYP2R1*), vitamin D level and associations with RA.

**Materials and Methods:**

Totally 76 participants (35 RA patients and 41 healthy controls) were enrolled from a case-control vitamin D and *VDR* gene polymorphisms study regarding age and vitamin D concentration. CpG islands in promoter regions of the *VDR, CYP24A1, CYP2R1* genes were chosen for DNA methylation analysis by means of pyrosequencing. Chemiluminescent microplate immunoassay was used to assess 25(OH)D serum levels. RA clinical data, i.e. the disease activity score C-reactive protein 28 (DAS28 – CRP) as well as patient-reported outcome questionnaires were recorded.

**Results:**

The study showed similar methylation pattern in the promoter regions of vitamin D pathway genes in RA and control group with *p*>0.05 (*VDR* gene 2.39% *vs.* 2.48%, *CYP24A1* gene 16.02% *vs.* 15.17% and *CYP2R1* 2.53% *vs.* 2.41%). *CYP24A1* methylation intensity was significantly higher in compare to methylation intensity of *VDR* and *CYP2R1* genes in both groups (*p*<0.0001). A tendency of higher vitamin D concentration in cases having methylated *VDR (*57.57±28.93 *vs.* 47.40±29.88 nmol/l), *CYP24A1 (*53.23±26.22 *vs.* 48.23±34.41 nmol/l) and *CYP2R1* (60.41±30.73 *vs.* 44.54±27.63 nmol/l) genes and a positive correlation between *VDR, CYP2R1* methylation intensity and vitamin D level in RA affected participants was revealed (*p*>0.05). A significantly higher *CYP24A1* methylation intensity (*p*=0.0104) was detected in blood cells of vitamin D deficient (<50 nmol/l) RA patients *vs.* vitamin D deficient controls.

**Conclusions:**

Our data suggests some indirect associations between DNA methylation status of vitamin D pathway genes and vitamin D level in RA.

## Santrauka.

**Įvadas.:**

Reumatoidinis artritas (RA) yra sudėtingos etiopatogenezės autoimuninė uždegiminė liga, nulemta daugelio rizikos veiksnių, kuriems priskiriamos ir epigenetinės modifikacijos. Manoma, kad RA yra susijęs su vitaminu D, kuris yra epigenetiškai aktyvus ir gali daryti įtaką tam tikrų genų DNR metilinimui. Šiuo tyrimu siekta įvertinti vitamino D atsako į genų (*VDR, CYP24A1, CYP2R1*) DNR metilinimo sąsajas su vitamino D kiekiu bei RA.

**Medžiaga ir metodai.:**

Į tyrimą iš viso įtraukti 76 tiriamieji (35 RA pacientai ir 41 kontrolinės grupės asmuo), pagal amžių ir vitamino D koncentraciją kraujo serume atrinkti iš *VDR* geno polimorfizmų atvejo kontrolės tyrimo. *VDR, CYP24A1, CYP2R1* genų promotorių CpG salų metilinimo analizė atlikta pirosekoskaitos metodu. Vitamino D koncentracijos kraujo serume tyrimas atliktas naudojant chemiliuminescencinę imuninę analizę. Surinkti RA klinikiniai duomenys: ligos aktyvumo indeksas vertinant 28 sąnarius (angl. *Disease activity score*, DAS28) ir būklės įsivertinimo klausimynų duomenys.

**Rezultatai.:**

Tyrimo metu buvo rastas panašus RA ir kontrolinės grupės tiriamųjų vitamino D apykaitos genų metilinimo lygis (*VDR* geno 2,39 % *vs* 2,48 %, *CYP24A1* geno 16,02 % *vs* 15,17 % ir *CYP2R1* geno 2,53 % *vs* 2,41 %). *CYP24A1* metilinimo intensyvumas buvo statistiškai reikšmingai didesnis, palyginti su *VDR* ir *CYP2R1* genų metilinimo intensyvumu abiejose grupėse (*p* < 0,0001).Tendencingai didesnė vitamino D koncentracija nustatyta RA tiriamiesiems, kurių genai buvo metilinti, palyginti su nemetilintais atvejais: *VDR* geno (57,57 ± 28,93 *vs* 47,40 ± 29,88 nmol/l), *CYP24A1* (53,23 ± 26,22 *vs* 48,23 ± 34,41 nmol/l), *CYP2R1* geno (60,41 *±* 30,73 *vs* 44,54 ± 27,63 nmol/l); *VDR* ir *CYP2R1* metilinimo intensyvumas teigiamai koreliavo su vitamino D koncentracija RA tiriamųjų grupėje (*p* > 0,05). Statistiškai reikšmingai didesnis *CYP24A1* metilinimo intensyvumas nustatytas periferinio kraujo ląstelėse tų RA pacientų, kuriems rastas vitamino D trūkumas (<50 nmol/l), palyginti su vitamino D stokojančiais sveikais asmenimis (*p* = 0,0104).

**Išvados.:**

Remiantis gautais duomenimis galima daryti prielaidą, kad vitamino D apykaitos genų metilinimo būklė yra netiesiogiai susijusi su vitamino D kiekiu sergant RA.

## Introduction

Rheumatoid arthritis (RA) is one of the most common autoimmune inflammatory arthritis with a complex pathogenesis mainly related to the breakdown of immune tolerance, autoantigen presentation with antigen specific T and B cells activation and aberrant production of inflammatory cytokines [1]. The disease manifests as synovial joint inflammation marked by infiltration of immune cells and damage to the extracellular matrix, leading to pain, disability, joint destruction and function loss [2]. It is known that RA development is associated with multiple risk factors that combine interaction between genetic predisposition, environmental risk and epigenetic changes [3]. However, the etiology and pathogenetic mechanisms of disease remain to be not fully unravelled thus demanding new researches, especially in the field of epigenetics.

Recently, epigenetic alterations were named as potential key players in pathogenesis of RA leading to heritable changes in gene activity and expression, without causing alterations in primary deoxyribonucleic acid (DNA) sequence thus regulating various cells’ behaviour [2, 4]. Changes of methylation pattern in peripheral blood T and B cells have been demonstrated in previous reports and suggest that epigenetic variation may mediate pathogenic activity of immune cells in RA [5-7]. These mechanisms lead to increased production of pro-inflammatory cytokines (such as interleukin (IL)-1β, IL-6, tumor necrosis factor (TNF)-α, etc.) and mediate development of chronic inflammation hereafter strengthening the hypothesis of altered DNA methylation signature in RA [3].

Furthermore, the environmental stress can also be reflected in genome as aberrant epigenetic marks, contributing to gene regulation and RA etiopathogenesis mechanisms [2]. Vitamin D is one of the potential environmental factors participating in RA course through the regulation and differentiation of the immune cells, performing its’ extra-skeletal anti-inflammatory, immuno-modulatory role [8-10]. The metabolism of vitamin D consists of multiple conversions mediated by cytochrome P450 (CYP) enzymes. The enzyme 25-hydroxylase (CYP2R1) converts cholecalciferol to 25-hydroxycholecalciferol (25(OH)D_3_), 1α-hydroxylase (CYP27B1) converts to active form 1.25-dihydroxyvitamin D (calcitriol) whilst 24-hydroxylase (CYP24A1) inactivates and decreases the calcitriol level either decreases the substrate available for its production [11]. The other important vitamin D signaling system player is the vitamin D receptor (VDR) which is expressed in multiple immune cells. It is also the key mediator in active vitamin D regulated gene expression [12]. Calcitriol interacts with the epigenome on multiple levels, e.g., binds the VDR and by the heterodimerized form further binds vitamin D response elements which lie in the promoter regions of vitamin D responsive genes and in this manner upregulates or suppresses DNA transcription [11, 13]. On the other hand, the above mentioned “vitamin D tool” genes coding VDR, CYP2R1, CYP27A1 and CYP24A1 tend themselves to be epigenetically regulated [13]. Promoter regions of these genes span the Cytosine-phosphate-Guanine (CpG) dinucleotide islands and thus can be silenced by DNA methylation [13, 14]. It is known that vitamin D deficiency is prevalent in RA patients, is associated with higher disease activity and the level appears to be significantly lower compared to healthy controls [15, 16]. Hypothesis of higher risk to develop RA in vitamin D deficient subjects has also been raised [17]. Therefore, since the attention to vitamin D role in RA is increasing and 25(OH)D signaling pathway has been poorly investigated in RA subjects, there is a need to analyze the methylation pattern of vitamin D metabolism genes in this group of patients.

The present study evaluated vitamin D pathway genes (*VDR, CYP2R1, CYP24A1*) methylation level in peripheral blood mononuclear cells of RA subjects and healthy controls in order to identify novel vitamin D-RA associated DNA methylation sites, and the relationship between selected genes methylation status with vitamin D level and RA clinical parameters.

## Materials and methods

### Study cohorts

Totally 76 participants (35 RA patients and 41 healthy control subject) were included from a case-control vitamin D and *VDR* gene polymorphisms study regarding age and vitamin D concentration [18]. All participants were enrolled at Vilnius University Hospital Santaros Klinikos (VUHSK) Rheumatology Centre after informed consent was obtained according to the permission of Vilnius Regional Biomedical Research Ethics Committee (Approval No. 158200-18/5-1037-533). RA diagnosis was established by ACR/EULAR 2010 rheumatoid arthritis classification criteria or by 1987 ACR classification criteria if diagnosed before year 2010 [19, 20]. Researchers collected sociodemographic (sex, age, body mass index, smoking status, etc.) and clinical data from all RA patients (disease duration, present treatment, disease activity score 28 (DAS28), C-reactive protein (CRP, mg/l), etc.). The DAS28 CRP was measured by a researcher rheumatologist and categorized accordingly as high (≥5.1), moderate (>3.2 to 5.1), low disease activity (2.6 to ≤3.2) or RA remission (<2.6) [21]. All eligible control group subjects were invited to participate at VUHSK Family Medicine Centre by a family doctor and further referred to Rheumatology Centre for informed consent form signing, data and blood samples collection.

All recruited subjects were adults over 18 years old. Subjects who did not meet the inclusion criteria were excluded (diagnosed cancer (<5 years), other autoimmune comorbidities, pregnancy, etc.) from the study. For biochemical tests and epigenetic analysis, blood samples from all study subjects were collected, coded and labeled as required. The research was carried out in accordance with the principles of the Declaration of Helsinki of 1975, revised in 2013.

### Cells preparation

Blood samples for DNA analysis from RA and control group were collected using BD Vacutainer® CPT^TM^ Mononuclear Cell Preparation Tube – Sodium Citrate vacutainers (8 mL) (BD Biosciences, Franklin Lakes, NJ, USA). Peripheral blood mononuclear cells (PBMCs) were prepared using manufacturer’s recommendations and standardized procedures: tubes with blood samples were centrifuged (Centrifuge – Heraeus Megafuge 8 Centrifuge, Thermo Scientific (TS) part of Thermo Fisher Scientific (TFS), Wilmington, DE, USA) by stages and sequential cell washing steps adding phosphate-buffered saline (PBS) (Biochrom, Berlin, Germany) were performed as recommended. Samples with prepared PBMCs were labelled and stored at –80°C temperature until DNA methylation analysis.

### DNA methylation analysis

All 76 samples selected for DNA methylation analysis were age, sex, and vitamin D matched. DNA isolation was performed using commercial GeneJET Genomic DNA Purification Kit (TFS) by manufacturer’s recommendations. Isolated DNA concentration and purity was measured by 260 / 280 and 260 / 230 ratios using NanoDrop® 2000 spectrophotometer (TS, TFS). Each subject DNA sample was bisulphate converted using EZ DNA Methylation™ Kit (Zymo Research, CA, USA) by manufacturer’s recommendations. Converted DNA samples were labelled and stored at –20°C. CpG islands in promoter regions of *VDR, CYP24A1, CYP2R1* genes were chosen for DNA methylation analysis. Primers for selected genes polymerase chain reaction (PCR) and pyrosequencing were designed using Ensembl data base (https://www.ensembl.org/index.html) and PyroMark Assay Design Software (version 2.0.1.15, Qiagen, 2008, Germany). PCR and pyrosequencing primers are listed in Supplementary Material, Table S1. PCR method was used to amplify modified DNA. PCR cycling conditions are shown in Supplementary Material, Table S2. The success of DNA amplification was assessed by 3% agarose gel electrophoresis. Methylation was evaluated in 10 *VDR* gene CpG positions, 7 *CYP24A1* and 6 *CYP2R1* CpG positions by means of pyrosequencing of PCR products and performed using PyroMark Q24 platform (Qiagen, Berlin, Germany) by manufacturer’s recommendations. Methylation intensity of each CpG site was generated using PyroMark Q24 Software (version 2.0.6, Qiagen, 2009, Germany) and assessed as percentage.

### Vitamin D evaluation

Blood samples for 25(OH)D measurement from RA and control group were collected using BD Vacutainer Serum Separator Tubes (5 mL) (BD Biosciences, New Jersey, USA) and evaluated at VUHSK Centre of Laboratory medicine using chemiluminescent microplate immunoassay (Architect ci8200, Abbott Laboratories, IL, USA), with the ability to detect 25(OH)D_3_ from 98.6% to 101.1% and 25(OH)D_2_ from 80.5% to 84.4%. Vitamin D concentration was classified as normal (≥75 nmol/l), insufficient (≥50–75 nmol/l) or deficient (<50 nmol/l) according to the Endocrine Society clinical practice guideline [22]. To avoid sources of bias blood samples collection was performed from late October until mid-May.

### Data statistical analysis

Descriptive statistics for demographic, clinical and biochemical variables was applied. Baseline methylation levels of *VDR, CYP24A1* and *CYP2R1*genes were calculated. After that, qualitative and quantitative methylation analysis of listed genes’ promoters CpG sites was performed. After Shapiro–Wilk normality test was applied, a parametric paired t-test or non parametric Mann–Whitney rank-sum test was performed for quantitative DNA methylation level (methylation intensity) analysis. To assess associations for qualitative DNA methylation level (methylation frequency) the Fisher’s exact or Chi-square test was used. Spearman rank-order correlation analysis was applied for demographic, clinical, biochemical parameters and *VDR, CYP24A1, CYP2R1* genes promoter’smethylation level.

Statistical analysis and data visualization was performed with R (Version 1.1.383, RStudio Inc, Boston, MA, USA), Microsoft Office Excel 2016 (Microsoft Corporation, Redmond, WA, USA) GraphPad Prism (version 8.0.1, GraphPad Software, San Diego, CA, USA) and ClustVis web tool [23]. Values were considered to be statistically significant at *p*<0.05.

## Results

### Main study population characteristics

From a case-control vitamin D and *VDR* gene polymorphisms study [18] a group of 35 RA patients and 41 healthy controls was selected for further DNA methylation analysis of vitamin D metabolism pathway genes. Average age of RA subjects at enrolment into the study was 51.63±10.45 years, mean disease duration 12.40±8.22 years. For comparison analysis, age, sex and vitamin D matched healthy controls with mean age of 50.78±11.85 years were enrolled. In order to precisely assess epigenetic differences in both groups, measured serum concentration of 25(OH)D in RA patients did not differ significantly in comparison with controls (50.89±29.54 *vs.* 56.05±28.67, *p*>0.05). The detailed descriptive analysis of anthropometric, sociodemographic and clinical data of study participants is presented in Table 1.

**Table 1.** Characteristics of study participants

Characteristics	RA, N=35	Healthy Controls, N=41	*p* value
**Sex (N, %)**			
Female	34 (97.1)	40 (97.5)	0.91
Male	1 (2.9)	1 (2.5)	
**Age (years)**	51.63±10.45	50.78±11.85	0.74
**BMI (kg/m** ^2^ **)**	25.60±4.39	26.22±5.51	0.99
**Smoking status (N, %)**			
Current smokers	3 (8.6)	2 (4.9)	0.54
Former smokers	4 (11.4)	8 (19.5)	
Never smokers	28 (80)	31 (75.6)	
**Disease activity (DAS28 CRP score)**	4.20±1.45		
High (N, %)	7 (20)		
Moderate (N, %)	20 (57.2)		
Low (N, %)	4 (11.4)	N.A.	N.A.
Remission (N, %)	4 (11.4)		
**Disease duration (years)**	12.40±8.22	N.A.	N.A.
**Vitamin D (nmol/l)**	50.89±29.54	56.05±28.67	
Deficiency (<50 nmol/l) (N, %)	16 (45.7)	17 (41.5)	0.2745
Insufficiency (≥50 -75 nmol/l) (N, %)	10 (28.6)	11 (26.8)	
Normal (≥75 nmol/l)(N, %)	9 (25.7)	13 (31.7)	
**CRP (mg/l)**	6.32±7.12	N.A.	N.A.

BMI: body mass index; CRP: C reactive protein; DAS28: disease activity score 28; RA: rheumatoid arthritis; N.A.; not applicable. Data represent mean ± standard deviation or N, %.

### DNA methylation analysis in peripheral blood mononuclear cells in RA patients and controls

In order to assess the methylation differences between the cases and controls, as well as its associations with RA clinical parameters and vitamin D level, DNA methylation level analysis of *VDR, CYP24A1, CYP2R1* promoters was performed by means of pyrosequencing.

#### VDR, CYP24A1, CYP2R1 methylation pattern

**Figure 1.**
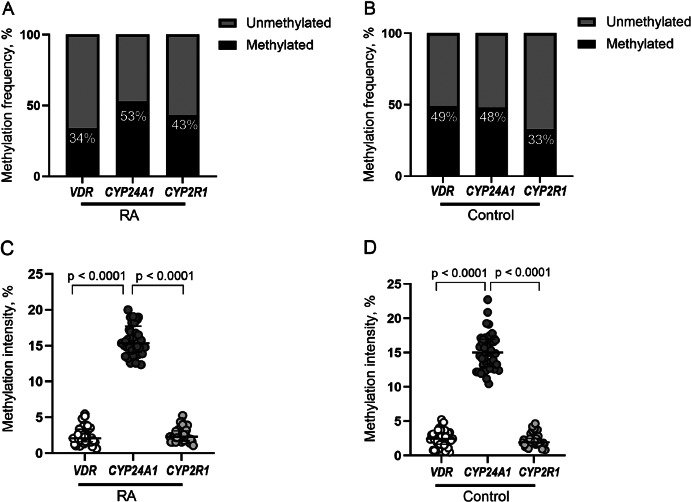
*VDR, CYP24A1* and *CYP2R1* promoter methylation frequencies (percentages) in RA (A) and healthy controls (B). Methylation intensity comparison of *VDR, CYP24A1* and *CYP2R1* genes promoters in RA patients (C) and healthy controls (D). RA: rheumatoid arthritis.

The mean methylation intensity of analyzed genes in RA *vs.* control group was as follows: *VDR* gene 2.39% *vs.* 2.48%, *CYP24A1* gene 16.02% *vs.* 15.17% and *CYP2R1* 2.53% *vs.*2.41%. According to the average methylation intensity of each gene promoter regions, the threshold of methylation level was specified, and methylated *vs.* unmethylated samples detected to access the methylation frequency of all analyzed CpG positions (Figure 1A and 1B). Although the differences between the cases and controls were statistically insignificant (*p*>0.05), *CYP24A1* methylation intensity was significantly higher compared to methylation intensity of *VDR* and *CYP2R1* genes promoters in both groups (*p*<0.0001) (Figure 1C and 1D).

**Figure 2.**
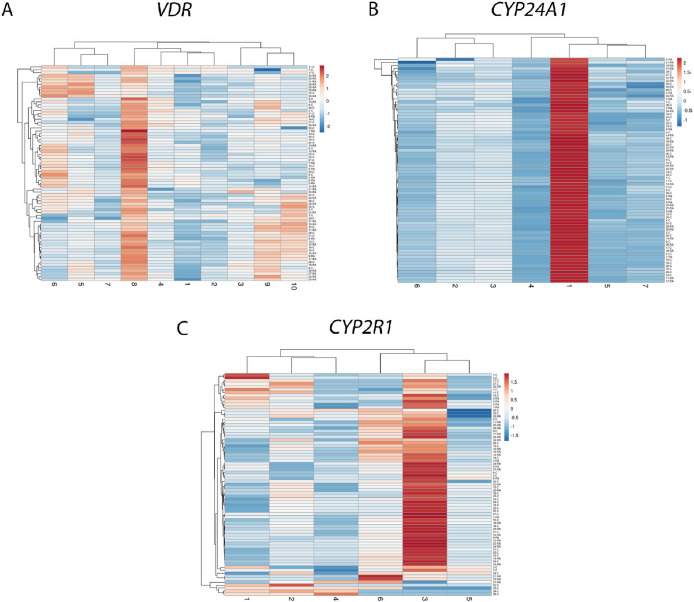
CpG methylation intensity comparison of *VDR* (A), *CYP24A1* (B), *CYP2R1* (C) promoters’ separate positions (X-axis) in RA and control group samples (Y-axis). Hierarchical clustering heatmap obtained using ClustVis web tool [23]. RA: rheumatoid arthritis. The blue-to-red color scale corresponds to the lowest and highest methylation intensity values.

The study also applied hierarchical clustering heatmap method to evaluate the methylation intensity of selected individual CpG positions located in promoter regions of *VDR, CYP24A1* and *CYP2R1* genes. No significant differences between RA and control group were detected (*p*>0.05). However, the comparison of separate CpG positions in RA revealed several significant differences. Significantly higher methylation intensity of *VDR* 8 CpG position compared to 1–4 and 7th positions was detected (1 *vs.* 8 *p*<0.0001; 2 *vs.* 8 *p*<0.0001; 3 *vs.* 8 *p*=0.0060; 4 *vs.* 8 *p*=0.0014; 7 *vs.* 8 *p*=0.0032). Also, the 1st CpG position of *CYP24A1* gene showed higher methylation intensity level in comparison with 2–7 positions (p < 0.0001) and 3rd position in comparison with 4–7 CpG positions (3 *vs.* 4 *p*<0.0001; 3 *vs.* 5 *p*=0.0005; 3 *vs.* 6 *p*=0.0079; 3 *vs.* 7 *p*=0.0020). Furthermore, 3rd CpG position of the *CYP2R1*gene was more intensively methylated than 1–5 positions (1 *vs.* 3 *p*=0.0019; 2 *vs.* 3 *p*=0.0286; 3 *vs.* 4 *p*=0.0003; 3 *vs.* 5 *p*=0,0035). Similar results were obtained from the control group analysis as well (Figure 2A, 2B and 2C). The graph with a more detailed information on mean methylation values of each gene CpG position and differences between the genes promoter regions in RA and controls is presented the Supplementary Material Figure S1 A and B.

Hereafter, associations between selected genes promoters’ methylation pattern and demographic features in RA group have been analyzed. *CYP24A1* methylation level positively correlated with age in RA patients (r=0.519, *p*=0.0017). Also, after comparing methylation frequencies, *CYP24A1* gene methylation was found to be significantly more frequent in older RA *vs.* younger participants (*p*<0.01). However, similar methylation changes were also seen in control group, possibly indicating universal age-related changes (*p*=0.01). No other significant associations between demographic parameters (i.e. sex, smoking status, etc.) and methylation pattern were discovered.

#### DNA methylation and RA clinical parameters

**Figure 3.**
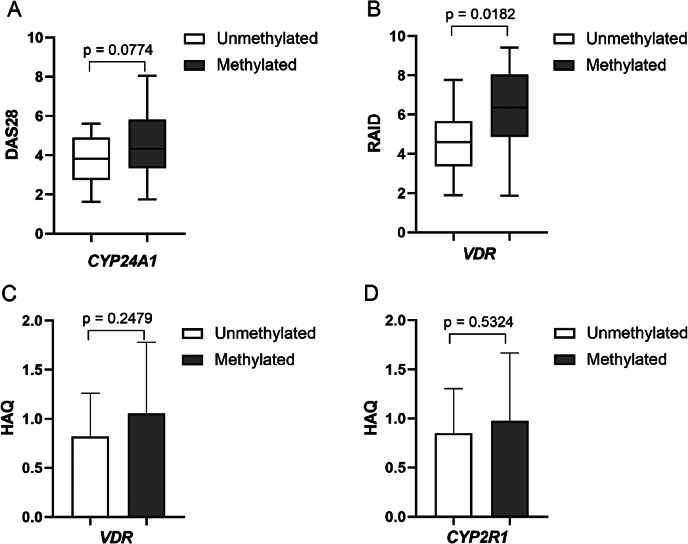
Comparison of cases with methylated *vs.* unmethylated *CYP24A1, VDR* and *CYP2R1* genes promoters and RA clinical parameters: DAS28 score (A), RAID score (B) and HAQ score (C). DAS28: disease activity score; HAQ: health assessment questionnaire; RA: rheumatoid arthritis; RAID: rheumatoid arthritis impact of disease.

The association of DNA methylation and RA disease clinical parameters was evaluated in the study. A tendency of higher RA DAS28 CRP disease activity score in *CYP24A1* methylated *vs.* unmethylated gene promoter RA cases (3.71±0.891 *vs.* 4.60±0.48, *p*=0.0774) was found (Figure 3A). However, the study found no significant associations between selected genes DNA methylation intensity and DAS28 CRP score in RA group. Nevertheless, a higher RAID score was significantly associated with *VDR* gene promoter methylation status (4.58±1.64 *vs.* 6.22±0.66, *p*=0.018) (Figure 3B). Also, *VDR*, as well as *CYP2R1*, methylated *vs.* unmethylated promoter cases had a higher HAQ score (0.82±0.20 *vs.* 1.05±0.23 and 0.85±0.19 *vs.* 0.97±0.12, respectively), however it was not statistically significant (Figure 3C). Other RA clinical variables, such as CRP, did not show any significant differences in respect of gene’s methylation level.

#### DNA methylation and vitamin D level

**Figure 4.**
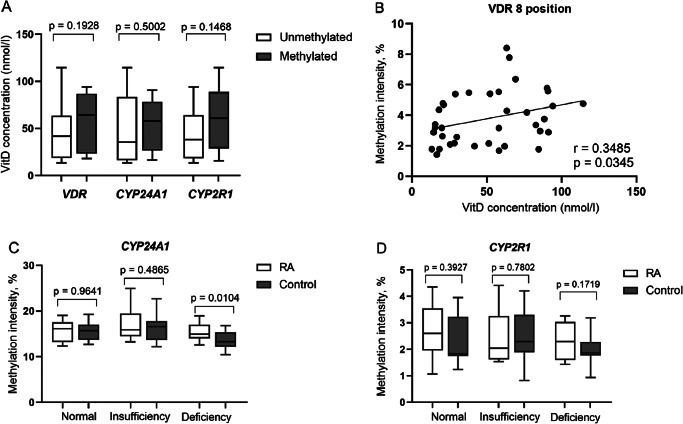
Vitamin D level (nmol/l) comparison by *VDR, CYP24A1* and *CYP2R1* genes promoter methylation frequency in RA subjects (A); *VDR* 8th CpG position methylation intensity (percentages) correlation with vitamin D level (nmol/l) (B); Comparison of methylation intensity of *CYP24A1* (C) and *CYP2R1* (D) promoters according to vitamin D level (nmol/l) in RA subjects and healthy controls. RA: rheumatoid arthritis; VitD: vitamin D

The study data showed higher vitamin D concentration in RA cases with methylated *vs.* unmethylated *VDR* (57.57±28.93 *vs.* 47.40±29.88 nmol/l), *CYP24A1* (53.23±26.22 *vs.* 48.23±34.41 nmol/l) and *CYP2R1*(60.41±30.73 *vs.* 44.54±27.63 nmol/l) gene promoters, indicating that DNA methylation pattern of afore mentioned genes tend to be associated with higher vitamin D level. The differences were not statistically significant (Figure 4A), however, methylation intensity analysis of separate CpG positions revealed a significant positive correlation of *VDR* 8^th^ position and vitamin D level (r=0.3485, *p*=0.0345) in RA (Figure 4B). Furthermore, methylation intensity of *VDR* and *CYP2R1* genes promoters positively correlated with vitamin D concentration in RA patients, though not significantly (*p*>0.05). Vitamin D deficient (<50 nmol/l) RA subjects revealed a significantly higher *CYP24A1* methylation intensity *vs.* vitamin D deficient controls (*p*=0.0104), thus indicating the disturbed vitamin D metabolism in RA (Figure 4C). Additionally, a higher *CYP2R1* methylation intensity was also revealed in vitamin D deficient, as well as normal vitamin D level RA *vs.* control group, respectively, however not significant (Figure 4D). The methylation level of selected genes promoters did not differ significantly according to vitamin D status (normal range (≥75 nmol/l) *vs.* insufficiency (≥50–75 nmol/l) or deficiency (<50 nmol/l) and vice versa) in RA group. The use of vitamin D supplementation also did not show any statistically significant differences between the groups.

## Discussion

The accumulating evidence indicates that epigenetic changes are stated as possible contributors in complex RA mechanisms, biomarkers for diagnosis or even therapeutic targets [3, 24]. Vitamin D acting as immunomodulator in various cell behavior mechanisms might be responsible for epigenetic modifications and consequently contribute to RA pathogenesis [12, 13, 25]. However, none of previously reported studies have analyzed DNA methylation pattern of vitamin D pathway genes in RA patients to date. The present study aimed to evaluate methylation level of *VDR, CYP24A1* and *CYP2R1* genes promoter regions in peripheral blood cells of RA and healthy controls in order to assess the differences between the groups, possible associations of epigenetic changes with RA clinical parameters and vitamin D level.

The study showed similar methylation pattern in the promoter regions of vitamin D pathway genes in RA and control group. Additionally, an overall low DNA methylation level was observed, except for *CYP24A1*, which revealed a significantly higher mean methylation intensity of approximately 15 to 17 percent in both groups. Similarly to our results, a study analysing *VDR* methylation in Behcet’s disease affected subjects and healthy controls reported no significant differences between the groups [26]. Our data is also consistent with the study conducted by Beckett et al. that showed low average methylation level of analyzed *CYP2R1, CYP24A1* and *VDR* genes [27]. A higher methylation level of *CYP24A1* gene promoter revealed in our study may be related to high prevalence of vitamin D deficiency in both study groups, RA and control, since CYP24A1 enzyme is responsible for degradation of calcitriol – an active form of vitamin D. On the other hand, low *CYP2R1* and *VDR* methylation intensity allows normal gene expression and indirectly indicates active vitamin D metabolism process.

Confirming the hypothesis, our study results showed a tendency of higher vitamin D concentration in RA patients with methylated *VDR, CYP24A1, CYP2R1* promoters and a positive correlation between *VDR, CYP2R1* methylation intensity and vitamin D level in RA. In accordance to our findings, Zhu H. and colleagues revealed that participants with severe vitamin D deficiency (25(OH)D ≤25 nmol/l) showed a pattern of reduced methylation of *CYP2R1, CYP24A1* and *DHCR7* genes in comparison with controls (25(OH)D >75 nmol/L) [28]. The study conducted by Beckett et al. also demonstrated a positive plasma 25(OH)D level correlation with *VDR* gene methylation level, meanwhile *CYP2R1* and *CYP24A1* methylation, in contrast to our results, negatively correlated with vitamin D concentration [27]. Also, in contrary to our results, a vitamin D (1100 IU/day) and calcium intervention trial identified that the average methylation of *CYP2R1* and *CYP24A1* genes was negatively associated with 12-month increase in serum 25(OH)D. Interestingly, non responders to vitamin D supplements at baseline had significantly higher *CYP2R1* and *CYP24A1* methylation levels compared to that in the responders thus predicting vitamin D response [29]. Regarding the results of present study and previous findings, associations demonstrated between 25(OH)D level and *CYP24A1, CYP2R1, VDR* promoters’ methylation support the relationship between vitamin D and the epigenome.

A more detailed analysis regarding categorized vitamin D level showed some significant findings between the groups. A noteworthy revelation of the present study is the observed higher methylation level of *CYP24A1* and *CYP2R1* genes promoters in vitamin D deficient (<50 nmol/l) RA participants *vs.* vitamin D deficient control group. Furthermore, RA patients with normal vitamin D concentration (≥75 nmol/l) presented higher *CYP2R1* methylation level *vs.* equivalent vitamin D level control subgroup. Although, the DNA methylation profile in RA tends to be globally hypomethylated rather than hypermethylated in PBMCs and RA fibroblast-like synoviocytes [30, 31], DNA methylation changes that are demonstrated in our study could prompt considerable reasons of higher vitamin D deficiency prevalence in RA subjects. This could be explained by hypoproduction of vitamin D metabolism enzymes induced by gene silencing, as mentioned before.

Despite the homogeneously formed RA and control groups regarding age and vitamin D level, the study showed no significant differences of vitamin D pathway genes between the groups. Notably, this suggests that the methylation pattern of vitamin D pathway genes is contingently associated with lower vitamin D level in RA. Also, it is known that the presence of 25(OH)D metabolism enzymes, except for VDR, is more relevant in other cells than PBMCs [12], thus prompting that the methylation level of selected genes may differ in various tissues. Furthermore, one of the potential drawbacks of our study was the limited sample size and overall high prevalence of suboptimal vitamin D level in both groups. The above mentioned factors could have affected the significance of analyzed data. Moreover, causal relationships between the variables could not be assessed due to the study design. Despite the careful technical procedures, the methylation status of the promoters was determined by analysis of selected CpGs, which might not completely reflect the methylation level of entire region. Also, other genes and other regulatory mechanisms involved in regulation of vitamin D pathway should be included in further studies of vitamin D role in RA.

In summary, a tendency of higher vitamin D concentration in cases with methylated *VDR, CYP24A1, CYP2R1* genes promoters and a positive correlation between *VDR, CYP2R1* methylation intensity and vitamin D level in RA affected participants was shown. Vitamin D deficient (<50 nmol/l) RA patients revealed a significantly higher *CYP24A1* methylation intensity *vs.* vitamin D deficient controls. The analyzed data indicate that the methylation pattern of vitamin D metabolism genes is indirectly associated with vitamin D level in RA. Therefore, the necessity of further investigations of vitamin D regulatory mechanisms in RA patients remains to be actual.

## Supplementary Material

https://www.journals.vu.lt/AML/article/view/24702/25627
